# A qualitative study on the working experiences of clinical pharmacists in fighting against COVID-19

**DOI:** 10.1186/s12913-021-07419-8

**Published:** 2022-01-05

**Authors:** Xiaojuan Wang, Xiali Yao, Xuedong Jia, Xiangfen Shi, Jie Hao, Yantao Yang, Gang Liu, Xiaojian Zhang, Shuzhang Du, Zhao Yin

**Affiliations:** 1 Department of pharmacy, The First Affiliated Hospital of Zhengzhou University, Zhengzhou, Henan China; 2 Department of Pharmacology, School of Basic Medical Sciences, Guizhou Medical University, Guiyang, Guizhou China

**Keywords:** Novel coronavirus, COVID-19, Clinical pharmacists, Working experiences, Qualitative study

## Abstract

**Background:**

The spread of coronavirus disease 2019 (COVID-19) has overwhelmed healthcare systems across the world. Along with the medical team, clinical pharmacists played a significant role during the public health emergency of COVID-19. This study aimed to explore the working experience of clinical pharmacists and provide reference for first-line clinical pharmacists to prepare for fighting against COVID-19.

**Methods:**

A qualitative study based on descriptive phenomenology was employed with face-to-face and audio-recorded interviews to study the working experience of 13 clinical pharmacists (including two clinical nutritional pharmacists). All interviews were transcribed verbatim, and the interview data were analyzed thematically using NVivo software.

**Results:**

Four themes emerged from interview data, including roles of clinical pharmacists, working experiences of clinical pharmacists, psychological feelings of clinical pharmacists, and career expectations of clinical pharmacists.

**Conclusions:**

The results contributed to a deeper understanding of the clinical pharmacists’ work experiences in COVID-19 and offered guidance to better prepare clinical pharmacists in participating in a public health crisis.

**Supplementary Information:**

The online version contains supplementary material available at 10.1186/s12913-021-07419-8.

## Background

In December 2019, a novel coronavirus (2019-nCoV) was first detected in Wuhan, Hubei Province, China, leading to a nationwide outbreak [1]. On February 11, 2020, the World Health Organization (WHO) officially named the Coronavirus Disease 2019 (COVID-19). Exactly 1 month later, on March 11, 2020, the WHO declared COVID-19 a pandemic. As of July 20, 2020, COVID-19 has caused 14,654,560 infections and 609,135 deaths globally. COVID-19 has become a public health event that requires worldwide attention and collaboration [2, 3].

Medical workers in China, including pharmacists, have been actively involved in preventing and treating COVID-19 [4]. Alongside nurses, physicians, and respiratory therapists, clinical pharmacists contribute to COVID-19 management by participating in inpatient rounds and providing drug information [5]. Pharmacists also play a crucial role by ensuring sufficient medication supply by managing critical drug shortages [6, 7]. During the outbreak, many COVID-19 treatment drugs lacked clinical evidence and needed to be supported by clinical trials [8]. This includes antiviral drugs, antimicrobial agents, hormones, traditional Chinese medicines, and other adjuvant therapies. Pharmacists can help to enroll infected patients in these studies [9].

In many cases, clinical pharmacy services were fully or partially withdrawn from the ward to reduce the risk of infection and to conserve the usage of personal protective equipment. Despite this, clinical pharmacists continued to support patient care in hospitals through the use of technology. The withdrawal of clinical pharmacy services, however, raises concern that the role of clinical pharmacists is still poorly recognized [10]. Clinical pharmacists took the principles of drug treatment, drug interactions, therapeutic drug monitoring (TDM), medication for special population and adverse drug reactions as the breakthrough points, and implemented “one-to-one” pharmaceutical care, which was “one person, one strategy” medication recommendations to solve the problem of drug treatment effectively and have a high degree of clinical acceptance [11, 12]. The expert consensus of the Chinese Medical Doctor Association (CMDA) proposed that in the treatment of COVID-19, clinical pharmacists who specialize in anti-infection should be involved. Research on the working experiences of clinical pharmacists in fighting COVID-19 deserves to be conducted [9]. Therefore, the objective of the present study is to summarize the roles, responsibilities, and work challenges of frontline clinical pharmacists during the COVID-19 outbreak and provide reference for improving pharmacy operations in fighting future public health emergencies.

## Methods

### Study design, theoretical framework, and participants

Two hospitals in the Henan province which are designated for COVID-19 patients were selected as study samples. Hospital A (the Sixth People’s Hospital of Zhengzhou, AKA Zhengzhou Infectious Disease Hospital, 420 beds) has 60 COVID-19 treatment beds designated for treating severe COVID patients. Hospital B (the First Affiliated Hospital of Zhengzhou University, 8500 beds) has 35 beds for non-severe COVID-19 patients. Pharmacists who participate in the COVID-19 patient care at these hospitals generally have more than 3 years of working experience in respiratory, cardiovascular, and nutrition support disciplines.

We used combination of purposive and snowball sampling methods in this study. The inclusion criteria were pharmacists who (1) have been working as full-time pharmacists in respiratory, cardiovascular, and nutrition disciplines for at least 3 years, (2) acquired with at least a bachelor’s degree in pharmacy, and (3) were willing to participate in the present study. We determined the number of required participants by interviewing pharmacists who met the inclusion criteria until the data were saturated and no new topics were generated. Ultimately, thirteen clinical pharmacists were included in the analysis. All audio recordings and transcripts were saved on a password-protected computer.

The study was designed to deeply describe pharmacists’ experiences using the phenomenological methodology. This type of design is usually appropriate when only a few previous studies and grounded theories describe the phenomenon in question. Therefore, the study does not have one strict theoretical framework as a starting point. However, given the aim of the study, results can be interpreted from the perspectives of pharmacists’ psychological feelings, preparedness, and professional identity. The study followed the Standards for Reporting Qualitative Research (SRQR) [13] reporting guideline. The interview data were thematically analyzed using NVivo software (Version 12) [14].

### Data collection

Semi-structured, in-depth, face-to-face interviews were conducted by three trained interviewers convenient for participants between April and May 2020. Each interview lasted for 20–60 min. All interviews were audio-recorded, and participants’ non-verbal behaviors were noted. The participants’ age, gender, years of work experience, education level, and professional titles were obtained during the start of the interview (Table 1).

**Table 1:** The demographics of clinical pharmacists

ID	Age	Gender	Work experience, years	Education level	Professional title
1	35	Male	9	Undergraduate	Pharmacist-in-charge
2	33	Male	11	Undergraduate	Pharmacist-in-charge
3	35	Female	12	Undergraduate	Pharmacist-in-charge
4	30	Female	5	Master	Pharmacist-in-charge
5	32	Female	5	Master	Pharmacist-in-charge
6	36	Female	10	Master	Associate chief pharmacist
7	40	Female	14	Maste	Associate chief pharmacist
8	36	Female	11	Master	Associate chief pharmacist
9	35	Female	9	Master	Pharmacist-in-charge
10	43	Female	21	Master	Associate chief pharmacist
11	38	Female	10	Master	Pharmacist-in-charge
12	31	Female	4	Master	Pharmacist-in-charge
13	40	Female	16	Doctor	Associate chief pharmacist

We created the interview questions by consulting relevant literature, seeking experts’ opinions, and selecting two pharmacists for pre-interview. These questions were: (1) As a clinical pharmacist, please tell me about your experiences fighting against COVID-19. (2) As a clinical pharmacist, what else do you think we can do in the future? (3) What challenges did you encounter? and (4) What external support have you received? Probing questions, such as “Please tell me more about that,” were used to enhance discussion depth (The detailed interview guide is presented as supplementary file 1: Box 1).

### Data analysis

**Fig. 1:**
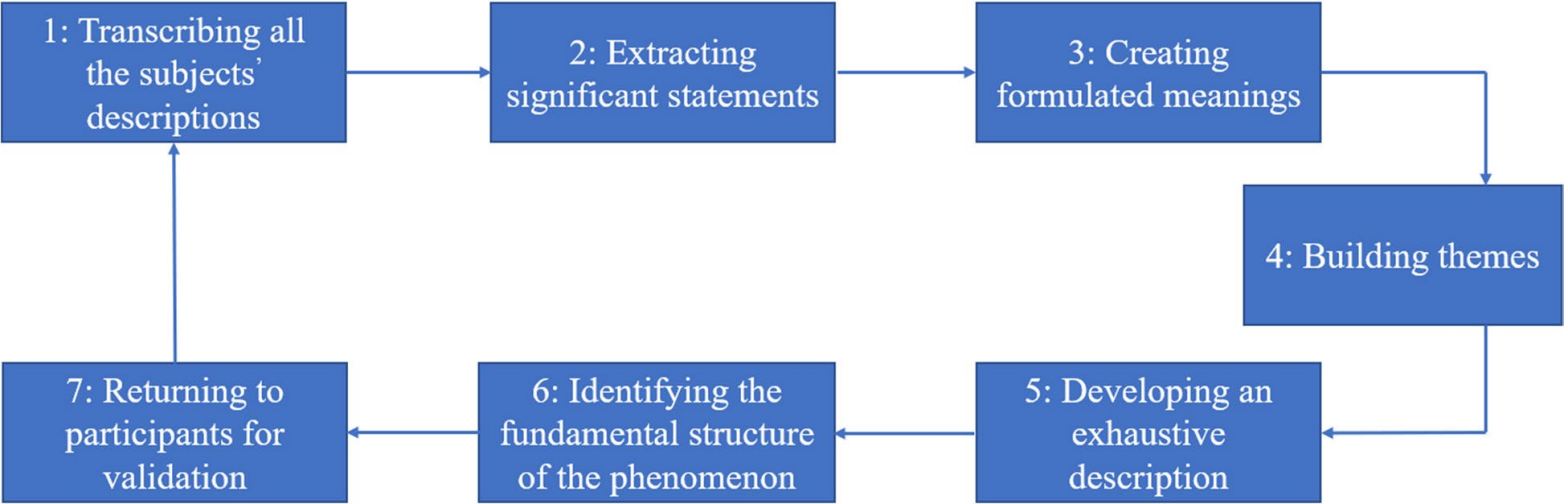
The whole process of Collaizzi seven-step approach

Thematic analysis was applied to understand the participants’ perceptions. The interviews were recorded digitally and subsequently transcribed verbatim. The transcripts were reviewed by two research members, checking for transcribing accuracy and consistency. All original recordings and transcriptions were in Chinese. The Chinese transcriptions were translated into English and back-translated into Chinese to ensure translation consistency. The transcripts were analyzed by bracketing data on preconceived ideas and strictly following the adapted Colaizzi seven-step analysis (Fig. 1). Two researchers coded the interviews independently using the NVivo software (Version 12). Extracting open topics, creating initial codes, and drafting categories or sub-categories were conducted in proper order. Representative quotations were selected by coders to present categories or sub-categories. Discussions were carried out whenever there were disagreements in the coding process until consensus was reached. A senior research expert was invited to check and revise the extracted codes, categories, and sub-categories. Colaizzi’s phenomenological method guarantees the authenticity of the collected experience of participants to adhere to scientific standards.

## Results

**Fig. 2:**
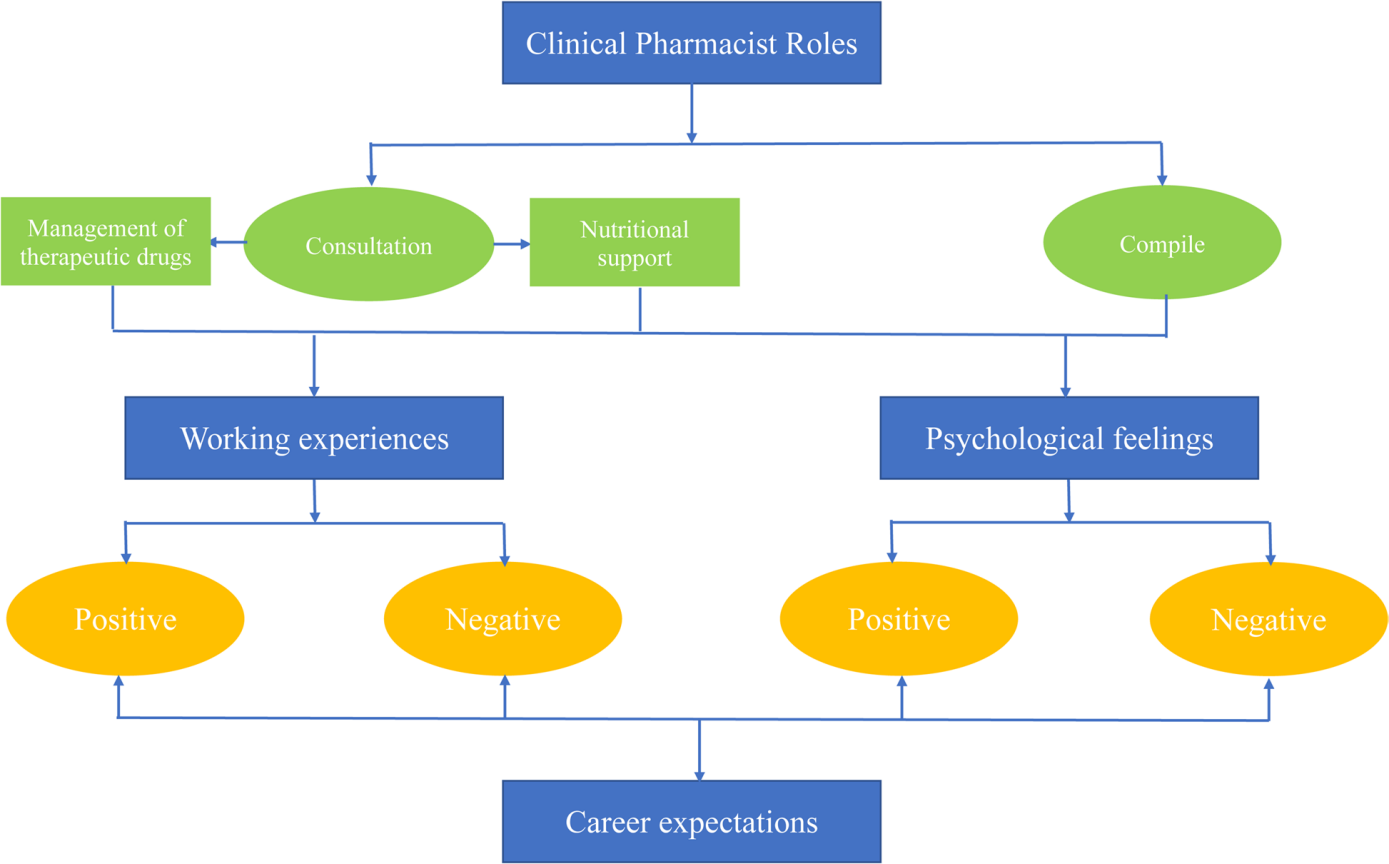
Four themes of clinical pharmacists’ experiences of combatting COVID-19

The following four themes were identified: clinical pharmacists’ roles, working experiences, psychological feelings, and career expectations. The results are described in Fig. 2.

### Theme 1: roles of clinical pharmacists

Clinical pharmacists’ roles in preventing and controlling COVID-19 mainly involved three aspects: (1) Participation in consultation and case discussion; (2) Nutritional support. (3) Preparation of pharmaceutical books related to epidemic prevention and control.

#### Participation in consultation and case discussion

The primary way clinical pharmacists participated in consultations was over the telephone when providers directly contacted the pharmacy department. Within the pharmacies, there were designated clinical pharmacists available for consultation. Clinical pharmacists of respiratory and cardiovascular specialties were also involved in the COVID-19 core expert treatment group. They conducted rounds and worked with clinicians in multi-disciplinary case discussions. Clinical pharmacists also joined the Henan COVID-19 Treatment Expert Group through the hospital telemedicine system.

*“Clinical pharmacists also participated in the telemedicine consultation organized by the Health Commission of Henan Province; the expert group came back daily in the face of over 130 designated hospitals in the province to give remote guidance to such hospitals. Then three issues of expert consensus in Henan Province were published.” [P13].*

Clinical pharmacists actively monitored patients’ drug therapies, adverse drug reactions, drug interactions, and adjustments to medication regimens for special populations.

*“As I worked on the anticoagulation, one patient suffered from massive bleeding after being discharged from hospital due to poor management of medical workers in the hospital in which the patient was admitted, and the nurse didn’t notice the coagulation indicators on that day when the patient was discharged from hospital (looking worried from her face). The coagulation indicator was extremely high on the day of discharge, and anticoagulants should have been given to the patient [P6].”*

*“Many critically ill patients were admitted in the hospital, some specialists were impossible to take overall consideration concerning the medication and interaction. Hence our pharmacists would be asked to adjust some regimens [P13].”*

Pharmacists played an indispensable role in the clinical setting. Their responsibilities included ensuring drug compatibility, proper preparation and administration, completing medication reconciliation, and integrating the clinical application of traditional Chinese medicine injections.

“*The severe patients with COVID-19 often had complicated infections and required combination therapies. We actively participated in the treatment regimen discussion. [P10].”*

*“Sometimes these patients were on dialysis and extracorporeal membrane oxygenation (ECMO) support, and these could affect drug concentration. We paid attention to adjusting drug dosing for these patients to ensure treatment efficacy and safety. [P9].”*

#### Nutritional support

Nutritional support serves as the basis of treatment for patients with COVID-19. Clinical nutrition pharmacists participated in the daily multi-disciplinary consultation. They monitored patients’ condition changes and assisted physicians in adjusting nutrition regimens.

“*I could offer great guidance and improve patients’ nutritional status by fully relying on my knowledge, and at least I could improve physical fitness even though no obvious improvement in one index was found [P11].”*

#### Participation in the development of pharmaceutical books related to epidemic prevention and control

Pharmacists prepared COVID-19 guidebooks for providers and hospital management. These books included chapters on hospital COVID prevention and control, a medication reference list, a quick search of therapeutic regimens, and medication information.

“*We have also compiled the quick reference list for medications, which was equivalent to the pocketbook, being easy for physicians to read. In addition, we participated in the verification and proofreading of Guidance Manual for COVID-19 Prevention and Control prepared by Clinical Pharmaceutical Society, Chinese Medical Association.” [P9]*

### Theme 2: working experience of clinical pharmacists

Clinical pharmacists’ working experiences showed dual characters. Most felt that their professional value was well recognized by the medical team. However, some pharmacists thought they were not fully integrated into the clinical practice due to insufficient work experience.

#### Positive working experience

##### The medical team had strong demand for clinical pharmacists

Medical departments and medical teams actively invited clinical pharmacists to join the diagnosis and treatment discussions. They had great demand for pharmacists in managing patients’ complex and quick-changing conditions.

*“The medical department made it clear that clinical pharmacists must be involved in the expert group when it is expected to be set up. Of course, this is a preliminary work; they may see the accumulation of the process for all these years and various works you participated in, and they accepted. They think it is useful to listen to the opinions of clinical pharmacists during multiple key disease consultations, including consultation of various difficult cases in the hospital [P10].”*

##### Cooperation with other medical workers

Departments with good cooperation with clinical pharmacists before the outbreak were more willing to work with pharmacists. These clinical teams have a more intuitive knowledge and understanding of the pharmacist teams’ professional quality through early collaboration and recognize clinical pharmacists’ values.

*“Furthermore, efforts for phone calls in practice have been made by us for many years, and they have got used to making a phone call and asked us to search information or for assistance if they have some problems, or they don’t have time to query [P2].”*

“*Under such circumstances, as usual, you just come and participate. Usually, we have done a good job, and basic work also has been done well, so it is recognized clinically [P13].”*

*“It is also increasingly dependent on our pharmacy; for sure, no matter what major you learn, you have what you focus on, you should think how it works, and how to let others accept us [P6]”.*

#### Negative working experience

##### Low degree of overall participation

The interview found that the degree of participation for clinical pharmacists was relatively low.

*“They (physicians) considered efficacy first, then safety; save life first, because it is most important. Sometimes, you have to consider efficacy first, regardless of big adverse reactions [P5].”*

*“What I considered at that time was nursing or doctors’ shortage; maybe nursing was scarcer. They focused more on people who needed to treat their diseases, and refined treatment and pharmaceutical care were considered next. Finally, we were not included in this respect (helpless smile) [P7].”*

##### Insufficient clinical work experience

Pharmacists had insufficient experience in recommending drug treatment. Most treatment recommendations were based on experiences against Severe Acute Respiratory Syndrome (SARS) in 2003 [15].

*“Traditional Chinese medicine injection was not necessarily safe, so we reminded doctors which ones were less safe. However, what was recommended at that time was determined by expert consensus, which maybe had no evidence-based basis [P6].”*

*“Since diagnosis and treatment guidelines were modified every week, and a large dose of ribavirin (more several times than daily dose) was recommended in the 5th Edition, which was very controversial. It remained controversial afterward (expression: memories). After 5 days, this dose was changed again and again at a national level; many attempts were made [P10]. “*

### Theme 3: psychological feelings of clinical pharmacists

Clinical pharmacists’ psychological feelings were complicated during the fight against COVID-19. Positive psychology primarily showed that as members of a medical team, pharmacists were very proud of their contributions to the country. They also felt delighted when patients got better and the number of infected persons dropped. Negative psychology mainly arose when patients’ conditions were complex and there was no effective therapy. Clinical pharmacists and the medical team felt stressed, anxious, and powerless when patients died.

#### A sense of professional identity and pride

With the deepening of work, pharmacists feel that their professional value is reflected. Therefore, pharmacists have a strong sense of professional pride.

*“I think I have a certain sense of professional identity and pride because it’s like a battle in which all the people are involved. Our power is minimal, but I can find some problems and solve some with my professional knowledge. Moreover, I think it is beneficial to give opinions about pharmacy from different perspectives [P10]. “*

*“The more you have done, the more confident you have. We can learn more, such as knowledge of other departments, which can be learned during the consultation, and learn from each other [P6].”*

#### A sense of the confidence

By participating in the treatment of patients on the spot and witnessing the recovery of patients, the professional pride of pharmacists has been further enhanced. In addition, positive media reports will also improve the confidence of pharmacists.

*“The happiest was that we received critical patients whose conditions were not good. But some people were cured and discharged from the hospital, and we were happy that those people recovered from acute disease. And I heard many reports from the front, including Wuhan. I was very excited and shed tears (tears in her eyes). I also feel it is very proud that you are involved, regardless of roles [P10].”*

#### A feeling of psychological pressure

Due to the seriousness of the epidemic, it is challenging for the medical team to treat patients, bringing tremendous psychological pressure to the pharmacists.

*“Then, as the disease progressed, what you have seen is that doctors were helpless. We were also helpless (lowered her head to silence and sigh); although I have been engaged in this for many years, it was rare that all of us were at a loss. The whole process was quite depressed; everyone was very nervous and tired, and got a lot of pressure; I almost had no rest [P11]. “*

### Theme 4: career expectations of clinical pharmacists

This pandemic exposed some problems related to pharmacists. These included insufficient knowledge reserve, little experience in emergency response, failure to work in heavy clinical tasks due to physical fitness, and failure to be competent at intricate clinical work due to inadequate professional skills. There are areas of future improvements for pharmacists and the profession.

#### Sufficient ability is the basis

Pharmacists are eager to participate more in clinical work, but sufficient ability is the basis for clinical work.

*“Then I think we should be involved deeper and broader, but this involvement also has a premise. That is, your professional ability should reach a certain level. As you know, for doctors, if you intend to be involved, they want you to solve the problem; if you cannot solve the problem, it doesn’t matter if you are there or not [P10].”*

#### Ability is not sufficient enough

At the frontline, pharmacists feel that their ability is not enough to help clinicians solve clinical problems. The reason may be that many pharmacists do not receive sufficient training and exercise, resulting in insufficient basic knowledge reserves and clinical experience.

*“Only about one-third of people throughout clinical pharmacy discipline can reach the current recognition, and the rest still need to be improved. Those with more advanced qualifications can reach this level because they have been involved in the clinic since 2000. This is a significanr point. Some other young ones have not yet reached this level [P13]. “*

*“Of course, there are a lot of things you don’t understand, and then you have to learn (the eyes are firm and powerful) [P6].”*

#### Clinical skills of pharmacists should be proved in the future

When clinicians encounter complicated patients, they hope that clinical pharmacists can provide timely therapeutic consultation. Therefore, pharmacists should strive to work on their clinical skills and prescribing support.

“*But when you encountered this situation, they didn’t care about (what your duty is), they thought you were a pharmacy worker, and you had come to help me. If I asked you for anything, you must immediately do it and provide a solution for me instantly. This is something that I think is very difficult. The reserve of my entire knowledge and the professional ability of emergency response is (not enough) [P13].* “

*“Because they didn’t have your help at the beginning, they didn’t think they needed you. If you have been providing help to them and you have achieved the effect they want, they will definitely want this kind of help [P6].”*

## Discussion

This qualitative study explored clinical pharmacists’ work and psychological experiences in epidemic prevention and control in Henan Province, China.

The results of this study reveal that the roles of Clinical pharmacists’ in preventing and controlling COVID-19 mainly include taking a role in consultation and case discussion, providing nutritional support for COVID-19 patients and compiling pharmaceutical books related to epidemic prevention and control. Similarly, the roles of pharmacists in COVID-19 in other countries have been reported [10, 16–18]. Pharmacists in Canada have actively participating in COVID-19 management, such as resolving drug shortages, developing treatment protocols, participating in rounds, interpreting lab results, recruiting patients for clinical trials, and providing antimicrobial stewardship [8]. Clinical pharmacists in the United States have long been recognized by other medical workers and considered valuable team members. Experience from one U.S. hospital identified that COVID-19 patients received an average of 19.8 different medications, and pharmacists made an average of eight interventions per patient. A panel of U.S. physicians and six clinical pharmacists co-authored the National Institute of Health (NIH) treatment guidelines for COVID-19 [19].

However, in China, the roles of pharmacists in medical treatment need to be strengthened [20, 21]. The China National Health Commission guidelines made no mention of pharmacists getting involved in the treatment plan. Clinical pharmacists have not fully exerted their professional [22] values due to the following factors of 1) the demand for physicians and nurses in a public health crisis is generally far more significant than pharmacists [23] and 2) the shortage of medical resources [24]. During the outbreak, resources such as masks and protective clothing were in short supply [25–27] and must be provided to doctors and nurses [28]. The entry of clinical pharmacists into patients’ rooms would be considered a waste of resources and increased infection risk [29, 30].

This study offers some suggestions to pharmacists and the profession to prepare for future public health events in China. First, we need to improve the pharmacy professional level and maximize clinical pharmacists’ roles [20, 31, 32]. Abundant professional knowledge is the basis for clinical pharmacists to gain a firm foothold in treatment teams [6]. Second, we need to provide platform support, spiritual support [30] and increase the clinical pharmacists’ participation in team-based care [33, 34]. The medical team increasingly recognizes the work of clinical pharmacists. Overall, the involvement of clinical pharmacists was not enough during the COVID outbreak in China. Only some clinical pharmacists in tertiary hospitals in China were exposed to the epidemic consultation work under the hospital platforms [35]. At the same time, leaders should encourage and support the work of clinical pharmacists. Third, we should pay attention to the mental well-being of clinical pharmacists [27]. There have been many published studies on the psychological stress of physicians and nurses. However, studies on the mental health of pharmacists are limited. Fourth, we should improve pharmacy emergency response plans and strengthen pharmacists’ emergency response capabilities [6, 36]. It is worth mentioning that several medical institutions in China have jointly issued emergency plans for hospital drug management under the epidemic of novel coronavirus pneumonia [9].

### Strengths and limitations

The present study is one of the first qualitative researches in Mainland China to explore the work experience of clinical pharmacists in the prevention and control of the COVID-19 epidemic. The results of this study provide a reference for the construction of pharmaceutical departments within medical institutions regarding their emergency plans for handling catastrophic public health events. The limit of this study is that the participants come from one province in central China. In the future, a nationwide in-depth research should be carried out.

## Conclusion

Clinical pharmacists have played several roles in combating COVID-19 in China. Compared with physicians and nurses, clinical pharmacists’ participation can be improved by addressing the problems uncovered from this study.

## Supplementary Information

**Additional file 1.**

## Data Availability

Data are available on reasonable request. The thematic data that support the findings of this present study are available from the corresponding author on reasonable request.

## Abbreviations

COVID-19: Coronavirus Disease 2019
ECMO: Extracorporeal Membrane Oxygenation
SARS: Severe Acute Respiratory Syndrome
CMDA: Chinese Medical Doctor Association
NIH: National Institute of Health
TDM: Therapeutic Drug Monitoring
