# Supplementary material for: A qualitative study on the working experiences of clinical pharmacists in fighting against COVID-19
Source: BMC Health Serv Res. 2022 Jan 5;22:28. doi: 10.1186/s12913-021-07419-8 (PMC8728479; doi:10.1186/s12913-021-07419-8)
Supplement: Supplementary file 1 — Additional file 1. [file 12913_2021_7419_MOESM1_ESM.docx]

**Box 1. Questions used in the interview guide**

**Q1:** **As a clinical pharmacist, please tell me about your experiences fighting against COVID-19?**

- When you knew about the outbreak, clinical pharmacists needed to join the medical staff to treat patients with COVID-19. What did you think at the time? what was your strongest feeling at that time?
- In addition to the ordinary work, what other work did you do at that time?

**Q2: As a clinical pharmacist, what else do you think we can do in the future?**

- What can you do to help the doctors and nurses?
- What do clinical pharmacists need to prepare for going to the front line?

**Q3: What challenges did you encounter?**

- Please tell me more about that how did you respond?

**Q4: What external support have you received?**

- What other support do you need?
